# Deucravacitinib for the treatment of moderate-to-severe palmoplantar pustulosis: Results from an early-terminated open-label trial

**DOI:** 10.1016/j.jdin.2025.09.015

**Published:** 2025-10-09

**Authors:** Ravi Ramessur, Zelma C. Chiesa Fuxench, Daniel Shin, Jennifer B. Mason, Anabel C. Mason, Maryte Papadopoulos, Bruna Galvao de Oliveira Wafae, Megan H. Noe, Joel M. Gelfand

**Affiliations:** a Department of Dermatology, Perelman School of Medicine, University of Pennsylvania, Philadelphia, Pennsylvania; b Department of Dermatology, Beth Israel Deaconess Medical Center, Boston, Massachusetts; c Department of Dermatology, Brigham and Women's Hospital, Harvard Medical School, Boston, Massachusetts

**Keywords:** clinical trial, deucravacitinib, palmoplantar pustulosis, psoriasis

*To the Editor:* Palmoplantar pustulosis (PPP) is a chronic inflammatory skin disease marked by recurrent pustules on the palms and soles. It causes significant impairment in health-related quality of life, pain, functional limitation, and psychological distress. Its clinical features and immunopathology differ in important ways from plaque psoriasis, but overlapping pathogenic pathways have been described, including involvement of interleukin (IL)-12, IL-23, and type I interferons.^1^

Existing treatment options for PPP are limited, and several biologic agents have failed to demonstrate benefit in clinical trials, possibly due to their large molecular weight, which may limit penetration into the epidermis, where cytokine networks drive PPP.^2^ Deucravacitinib, a small molecule TYK2 inhibitor, is approved for psoriasis. Its favorable safety profile, oral administration, and its modulation of IL-12, IL-23, and type I interferons make it a promising investigational agent for PPP.^3^

We conducted a single-arm, open-label, investigator-initiated trial (NCT05710185) to assess deucravacitinib 6 mg once daily in adults with moderate-to-severe PPP. The primary endpoint was the proportion of participants achieving at least 50% improvement in the Palmoplantar Psoriasis Area and Severity Index-50 at week 16. Secondary outcomes included change in Dermatology Life Quality Index, EuroQol Five-Dimension Questionnaire index, and visual analog scale (VAS), static Physician Global Assessment, and patient-reported pain and itch. The trial used a Simon’s two-stage design, with an interim analysis at 8 participants and a potential total sample of 18.^4^ The full methodology and outcome definitions are available in the Supplementary Appendix, available via Mendeley at https://data.mendeley.com/preview/8kzs4tcy8s?a=3166c2c6-ed83-4b7c-92f2-642c94a50db7.

The study was terminated early by the funder after 3 participants were enrolled. Key clinical and patient-reported outcomes are summarized in Table I. Participant baseline characteristics, detailed results, and participant clinical photographs are available in the Supplementary Appendix, available via Mendeley at https://data.mendeley.com/preview/8kzs4tcy8s?a=3166c2c6-ed83-4b7c-92f2-642c94a50db7. All had confirmed PPP and failed topical therapies, and 1 had extensive prior use of systemic and biologic treatments. One patient withdrew at week 12 (due to infection), 1 patient withdrew at week 16 (lack of efficacy), and 1 participant completed the full treatment period.

**Table I:** Longitudinal ppPASI scores for all 3 participants over the treatment course

Participant	ppPASI baseline	ppPASI week 16	DLQI (baseline → W16)	EQ-VAS (baseline → W16)	PGA at W16/last visit
1	28.8	26.5	16 → 19	75 → 50	3
2	18.8	13.7	9 → 7	81 → 85	2
3∗	15.2	12.7 (W8)	23 → 22 (W8)	0 → 50 (W8)	2 (W8)

*DLQI*, Dermatology Life Quality Index; *EQ-VAS*, EuroQol Five-Dimension Visual Analogue Scale; *PGA*, Physician Global Assessment; *ppPASI*, Palmoplantar Psoriasis Area and Severity Index.

∗ Participant 3 withdrew early following a serious adverse event and completed follow-up through week 8 only.

None of the 3 participants achieved the primary endpoint of Palmoplantar Psoriasis Area and Severity Index-50 at week 16. Patterns of response varied between participants, with no clear trend of improvement (Fig 1). At week 16, Dermatology Life Quality Index decreased from 16 to 13 (mean) in 2 participants. The EuroQol Five-Dimension Questionnaire index improved from 0.443 to 0.794 and the EuroQol Five-Dimension Visual Analogue Scale from 52.0 to 67.5. Pain and itch VAS scores fluctuated, with no consistent trend.

**Fig 1:**
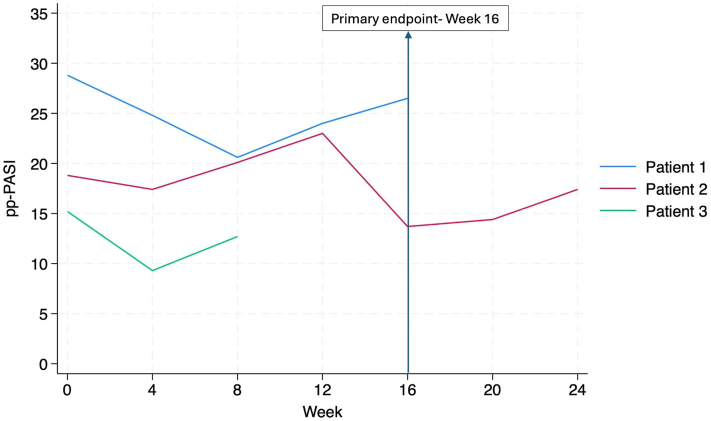
Longitudinal ppPASI scores by participant. *ppPASI*, Palmoplantar Pustulosis Area and Severity Index.

This small early-terminated trial did not find evidence supporting the efficacy of deucravacitinib in PPP at the standard psoriasis dose. While 1 participant reported improved perceived health, this was not reflected in objective measures. A previous retrospective series of 5 PPP patients treated with deucravacitinib reported mixed outcomes, consistent with the variability observed in this study.^5^

While the results may be inconclusive given the early termination before the planned futility analysis, no clinical responses were observed. These results underscore the need for larger, adequately powered studies to clarify the therapeutic potential of TYK2 inhibition in PPP. Further research is warranted to determine whether TYK2 inhibition for PPP may benefit selected subgroups or require alternative dosing strategies.

### Declaration of generative AI and AI-assisted technologies in the writing process

AI was not used in the conduct of this study or writing of the manuscript.

## Conflicts of interest

Dr Chiesa Fuxench has received research grants from Lilly, LEO Pharma, Brexogen, Regeneron, Sanofi, Tioga, and Vanda for work related to atopic dermatitis and from Menlo Therapeutics and Galderma for work related to prurigo nodularis. She has also served as a consultant for the Asthma and Allergy Foundation of America, the National Eczema Association, AbbVie, Incyte Corporation, and Pfizer and received honoraria for CME work in atopic dermatitis sponsored by education grants from Regeneron/Sanofi and Pfizer and from Beiersdorf for work related to skin cancer and sun protection. Dr de Oliveira Wafae is an investigator for AbbVie, Eli Lilly, Incyte, Moonlake, Prometheus, Avalo, Sanofi, Insmed, UCB, and Bristol Myers Squibb. Dr Noe previously served as a consultant for Boehringer Ingelheim, Argenx, and Takeda and received research grants from Boehringer Ingelheim and Bristol Myers Squibb. Dr Noe is currently an employee of Sanofi and receives a salary. Dr Gelfand is a consultant for AbbVie, Artax (DSMB), Bristol Myers Squibb, Boehringer Ingelheim, Celldex (DSMB), FIDE (which is sponsored by multiple pharmaceutical companies), GSK, Inmagene (DSMB), Lilly, Leo, Moonlake (DSMB), Janssen Biologics, Novartis Corp, UCB (DSMB), Neuroderm (DSMB), Oruka, Inc, Teva (DSMB); receives research grants (to the Trustees of the University of Pennsylvania) from Amgen, Bristol Myers Squibb, and Pfizer Inc; and received payment for continuing medical education work related to psoriasis that was supported indirectly by pharmaceutical sponsors. Dr Gelfand is a deputy editor for the Journal of Investigative Dermatology, receiving honoraria from the Society for Investigative Dermatology; is chief medical editor for Healio Dermatology (receiving honoraria); and is a member of the Board of Directors for the International Psoriasis Council and the Medical Dermatology Society, receiving no honoraria. Drs Papadopoulos, A. C. Mason, J. B. Mason, Shin, and Ramessur have no conflicts of interest to declare.
